# MEK-ERK signaling is a therapeutic target in metastatic castration resistant prostate cancer

**DOI:** 10.1038/s41391-019-0134-5

**Published:** 2019-02-25

**Authors:** Nicholas G. Nickols, Ramin Nazarian, Shuang G. Zhao, Victor Tan, Vladislav Uzunangelov, Zheng Xia, Robert Baertsch, Elad Neeman, Allen C. Gao, George V. Thomas, Lauren Howard, Amanda M. De Hoedt, Josh Stuart, Theodore Goldstein, Kim Chi, Martin E. Gleave, Julie N. Graff, Tomasz M. Beer, Justin M. Drake, Christopher P. Evans, Rahul Aggarwal, Adam Foye, Felix Y. Feng, Eric J. Small, William J. Aronson, Stephen J. Freedland, Owen N. Witte, Jiaoti Huang, Joshi J. Alumkal, Robert E. Reiter, Matthew B. Rettig

**Affiliations:** 1 Department of Radiation Oncology, VA Greater Los Angeles Healthcare System, Los Angeles, CA 90073 USA; 2 Department of Radiation Oncology, David Geffen School of Medicine at University of California Los Angeles, Los Angeles, CA 90095 USA; 3 Department of Urology, David Geffen School of Medicine at University of California Los Angeles, Los Angeles, CA 90095 USA; 4 Division of Dermatology/Medicine, David Geffen School of Medicine at University of California Los Angeles, Los Angeles, CA 90095 USA; 5 Department of Radiation Oncology, University of Michigan, Ann Arbor, MI 48109 USA; 6 Department of Medicine, Division of Medical Oncology, Rutgers Robert Wood Johnson Medical School, Piscatway, NJ 08854 USA; 7 Center for Biomolecular Science and Engineering, Jack Baskin School of Engineering, University of California, Santa Cruz, Santa Cruz, CA 95064 USA; 8 Department of Molecular Microbiology & Immunology, Computational Biology Program, Oregon Health and Science University, Portland, OR 97239 USA; 9 Division of Hematology-Oncology/Medicine, David Geffen School of Medicine at University of California Los Angeles, Los Angeles, CA 90095 USA; 10 Department of Urology, School of Medicine, University of California Davis, Sacramento, CA 95817 USA; 11 Department of Pathology and Laboratory Medicine, Oregon Health and Science University, Portland, OR 97239 USA; 12 Department of Biostatistics and Bioinformatics, Duke University School of Medicine, Durham, NC 27710 USA; 13 Division of Urology, Durham Veterans Affairs Medical Center, Durham, NC 27705 USA; 14 Department of Biomolecular Engineering, University of California Santa Cruz, Santa Cruz, CA 95064 USA; 15 Division of Hematology and Oncology, Department of Medicine, University of California San Francisco, San Francisco, CA 94143 USA; 16 Vancouver Prostate Centre, Department of Urologic Sciences, University of British Columbia, Vancouver, BC V6T 1Z3 Canada; 17 VA Portland Health Care System, Portland and Knight Cancer Institute, Oregon Health and Science University, Portland, OR 97239 USA; 18 Department of Medicine, Oregon Health and Science University, Portland, OR 97239 USA; 19 Department of Radiation Oncology, University of California San Francisco, San Francisco, CA 94143 USA; 20 Division of Urology, VA Greater Los Angeles Healthcare System, Los Angeles, CA 90073 USA; 21 Division of Urology, Cedars-Sinai Medical Center, Los Angeles, CA 90048 USA; 22 Department of Microbiology, Immunology, and Molecular Genetics, David Geffen School of Medicine at University of California Los Angeles, Los Angeles, CA 90095 USA; 23 Department of Pathology, School of Medicine, Duke University, Durham, NC 27710 USA; 24 Division of Hematology-Oncology, Department of Medicine, David Geffen School of Medicine at University of California Los Angeles, Los Angeles, CA 90095 USA; 25 Department of Medicine, VA Greater Los Angeles Healthcare System, Los Angeles, CA 90073 USA

**Keywords:** Cancer genetics, Cancer therapy, Urological cancer, Prostate cancer, Prostate cancer

## Abstract

**Background:**

Metastatic castration resistant prostate cancer (mCRPC) is incurable and progression after drugs that target the androgen receptor-signaling axis is inevitable. Thus, there is an urgent need to develop more effective treatments beyond hormonal manipulation. We sought to identify activated kinases in mCRPC as therapeutic targets for existing, approved agents, with the goal of identifying candidate drugs for rapid translation into proof of concept Phase II trials in mCRPC.

**Methods:**

To identify evidence of activation of druggable kinases in these patients, we compared mRNA expression from metastatic biopsies of patients with mCRPC (*n* = 101) to mRNA expression in localized prostate from TCGA and used this analysis to infer differential kinase activity. In addition, we assessed the differential phosphorylation levels for key MAPK pathway kinases between mCRPC and localized prostate cancers.

**Results:**

Transcriptomic profiling of 101 patients with mCRPC as compared to patients with localized prostate cancer identified evidence of hyperactive ERK1, and whole genome sequencing revealed frequent amplifications of members of the MAPK pathway in 32% of this cohort. Next, we confirmed elevated levels of phosphorylated ERK1/2 in castration resistant prostate cancer as compared to untreated primary prostate cancer. We observed that the presence of detectable phosphorylated ERK1/2 in the primary tumor is associated with biochemical failure after radical prostatectomy independent of clinicopathologic features. ERK1 is the immediate downstream target of MEK1/2, which is druggable with trametinib, an approved therapeutic for melanoma. Trametinib elicited a profound biochemical and clinical response in a patient who had failed multiple prior treatments for mCRPC.

**Conclusions:**

We conclude that pharmacologic targeting of the MEK/ERK pathway may be a viable treatment strategy for patients with refractory metastatic prostate cancer. An ongoing Phase II trial tests this hypothesis.

## Introduction

There is an unmet need for new treatments for metastatic castration resistant prostate cancer (mCRPC) that is resistant to abiraterone acetate or enzalutamide. With adequate pre-clinical and clinical rationale, targeted therapeutic strategies beyond hormonal manipulation of the androgen receptor (AR) axis might be applied in this disease state. Small molecule kinase inhibitors are effective against multiple cancers [1]. In many cases, mutations in the kinase itself, or an upstream regulator, render these kinases constitutively active. However, identification of activating mutations in druggable kinases in mCRPCs is rare [2, 3]. Targeting of hyperactive wild-type kinases that nevertheless promote disease progression may still be a viable strategy [4–6]. Hyperactive kinase activity in prostate cancers can be inferred through downstream transcriptomic signatures and phosphoproteomics [6].

In the current study, our objective was to identify kinases activated in mCRPC that are targetable by existing, approved drugs, with the goal to identify candidate drugs for rapid translation into proof of concept Phase II trials in mCRPC. Virtual Inference of Protein-activity by Enriched Regulon (VIPER) analysis comparing mRNA expression signatures from 101 mCRPC tumors acquired as part of a multi-institutional trial [2, 7] to localized prostate adenocarcinoma (TCGA) identified ERK1 (MAPK) as a potential kinase target in mCRPC. ERK1 is druggable with trametinib, an inhibitor of MEK1/2, currently approved for melanoma. Prior reports demonstrate that ERK1/2 is phosphorylated in mCRPC at high frequencies despite a paucity of activating mutations in the principal proto-oncogenes in the pathway [8, 9]. Whole genome sequencing on this same cohort of metastatic tumors from patients with mCRPC was recently published, and consistent with prior sequencing reports of mCRPC metastases, there were infrequent activating mutations in genes within the MAPK pathway. However, we interrogated this sequencing data to detect amplification of selected MAPK pathway genes and found amplifications in 32% of this cohort. We also confirm ERK1/2 phosphorylation is characteristic of CRPC and report ERK1/2 phosphorylation in localized prostate cancers correlates with disease recurrence after surgery. In the clinic, we observed efficacy of trametinib monotherapy in a patient who failed multiple prior treatments for mCRPC. Our data suggest pharmacologic targeting of the MEK/ERK pathway may be a viable strategy for patients with refractory mCRPC. These results are rationale for an ongoing Phase II trial of MEK1/2 inhibitor trametinib in patients with mCRPC (NCT02881242).

## Materials and methods

### Metastatic biopsies of mCRPC, DNA, and RNA sequencing

The acquisition of mCRPC tumor biopsies, DNA whole genome, and RNA-sequencing were part of a multisite prospective IRB-approved trial (NCT02432001) described in detail elsewhere [2, 7]. Briefly, after obtaining signed informed consent, image guided biopsies were obtained from metastases (43% bone, 39% lymph node, 11% liver, 8% other site) from patients with mCRPC (27% after abiraterone, 17% after enzalutamide, 20% after both). Biopsies were embedded in OCT and snap frozen. Laser capture microdissection was used to enrich for tumor content. DNA and RNA was isolated and sequenced as previously described [2], with sequencing from 101 patients used in the present analysis. Amplification was defined as copy number ≥ 3 through methods as previously described [2, 7].

### VIPER analysis

VIPER was used to identify evidence of activation of selected protein kinase pathways from RNA-seq data sets as previously described [10]. Differential kinase activity was inferred between the two groups of samples (mCRPCs and primary prostate adenocarcinoma from TCGA) based on gene expression changes attributable to the kinases and downstream transcription factors. VIPER scores were computed and visualized using TumorMap [11].

### Immunohistochemistry

Tissue microarrays (TMAs) were constructed from prostatectomy specimens from patients without prior local or hormonal therapy and included separate arrays consisting of primary tumor, benign tissue adjacent to primary tumor, and normal prostate tissue separate from tumor foci. A CRPC TMA was constructed from tissue acquired from palliative transurethral resections in patients with CRPC after prior treatment with ADT alone. TMAs were stained for phosphorylation of residues Thr202/Tyr204 with an anti-ERK antibody (Cell Signaling Technology (catalog #4376). To compare CRPC, primary prostate cancer, and benign tissue, H-scores were calculated based on intensity of cellular staining and percent positive cells [12] and comparisons made between groups by Kruskal–Wallis test. A separate TMA was constructed from radical prostatectomy specimens from patients with localized prostate cancer who underwent curative intent radical prostatectomy between 1994 and 1999 without prior therapy, with detailed long-term clinical follow-up. These specimens were scored as positive or negative for any phosphorylated ERK1/2 staining. Hazard ratios for biochemical failure were calculated and adjusted for clinicopathologic features known to affect risk of recurrence.

### Phosphoproteomics

An existing, published phosphoproteomic database of mCRPC and localized treatment-naive prostate cancer tissue containing a mixture of tumor and benign gland was interrogated for known ERK1/2 kinase targets [6]. Data were filtered for a false discovery rate cutoff of 0.05, at least a 4-fold difference between mCRPC and benign, and the presence of a known curated function on phosphosite.org.

## Results

**Fig. 1:**
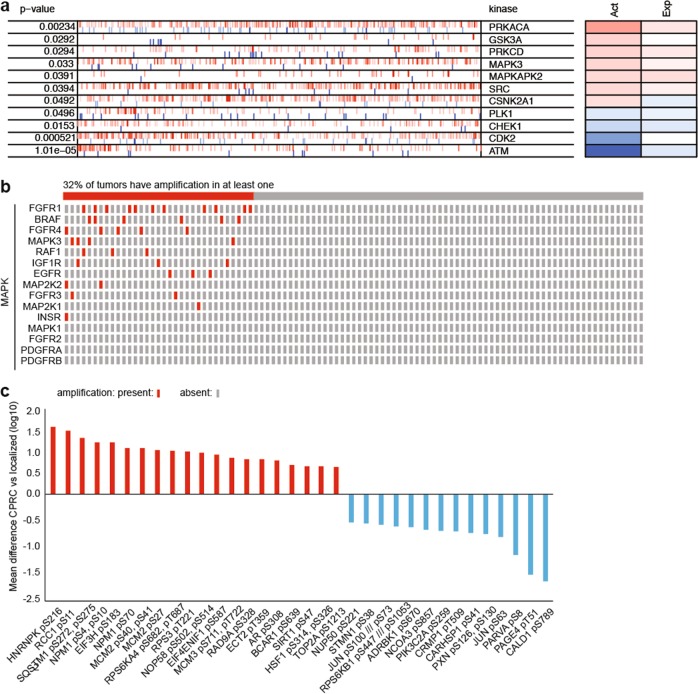
**a** Inferred kinase activation between mCRPC versus localized prostate adenocarcinoma (TCGA) by VIPER. The most activated (red) and repressed (blue) in the mCRPCs compared with localized prostate cancers (TCGA) with *p* < 0.05. Tick marks represent kinase targets projected onto the gene expression signature. Act: inferred differential activity; Exp: differential expression. MAPK3 = ERK1. **b** Presence or absence of amplification in selected MAPK pathway related genes in these patients. **c** ERK1/2 kinase targets differentially phosphorylated between mCRPC and treatment-naive localized prostate tissue. Data was filtered for a false discovery rate cutoff of 0.05, at least a 4-fold difference, and the presence of curated function on phosphosite.org

We sought to identify kinases activated in mCRPC that could be targeted by existing, approved drugs, with the goal of identifying candidate drugs for rapid translation into proof of concept Phase II trials in mCRPC. We employed Virtual Inference of Protein-activity by Enriched Regulon (VIPER) analysis on 53 kinases to identify potential kinase targets by virtue of their inferred activation in mCRPC [10]. VIPER scores were determined using mRNA expression signatures from 101 biopsies of CRPC metastases acquired as part of a multi-institutional trial [2, 7] to that of localized prostate cancer (TCGA) (Supplementary Table 1). This analysis identified 7 kinases with inferred activation significantly higher, and 4 lower, in mCRPC versus primary prostate cancers (Fig. 1a). Of the seven kinases with increased inferred activation, MAPK3 (ERK1) and SRC (c-SRC) are potentially actionable by approved drugs (e.g., trametinib and dasatanib, respectively). However, dasatanib, which inhibits SRC, was unsuccessful in a prior trial in mCRPC [13], so we focused on ERK1.

Given that point mutations in genes of MAPK pathway members have been rarely identified in patients with mCRPC [2, 3, 9], we hypothesized that amplifications of MAPK pathway genes [14, 15] or genes coding for proteins that have been shown to activate the MAPK pathway in mCRPC [16] would be more frequent in mCRPCs than in localized prostate cancers. The whole genome sequencing data [2] for the same patient cohort used to determine the VIPER scores was queried for any amplifications (focal or broad) of selected genes (Fig. 1b). Overall, more than 32% of the mCRPCs had amplifications [2] in one or more MAPK related genes, including amplifications of FGFR1 in 10% and BRAF in 6%.

We next sought to identify putative ERK kinase targets in mCRPC. ERK1 and ERK2 have similar substrate phosphorylation motifs [17]. We previously reported the phosphoproteome of mCRPC using phosphopeptides isolated from benign and cancerous prostates and mCRPC tissues via label-free mass spectroscopy [6]. We interrogated this existing dataset for known ERK1/2 substrates to identify potential phosphorylation targets of ERK1/2. Our phosphoproteomics study identified a total of 34 ERK1/2 kinase substrates and compared their phosphorylation rates in the metastatic tissues to those in the primary prostate tissues (Fig. 1c and Supplementary Table 2). Twenty proteins were found to be over-phosphorylated in metastatic tissues (Fig. 1c), of which, some were previously reported to be overexpressed in CRPC compared to hormone-sensitive tumors.

**Fig. 2:**
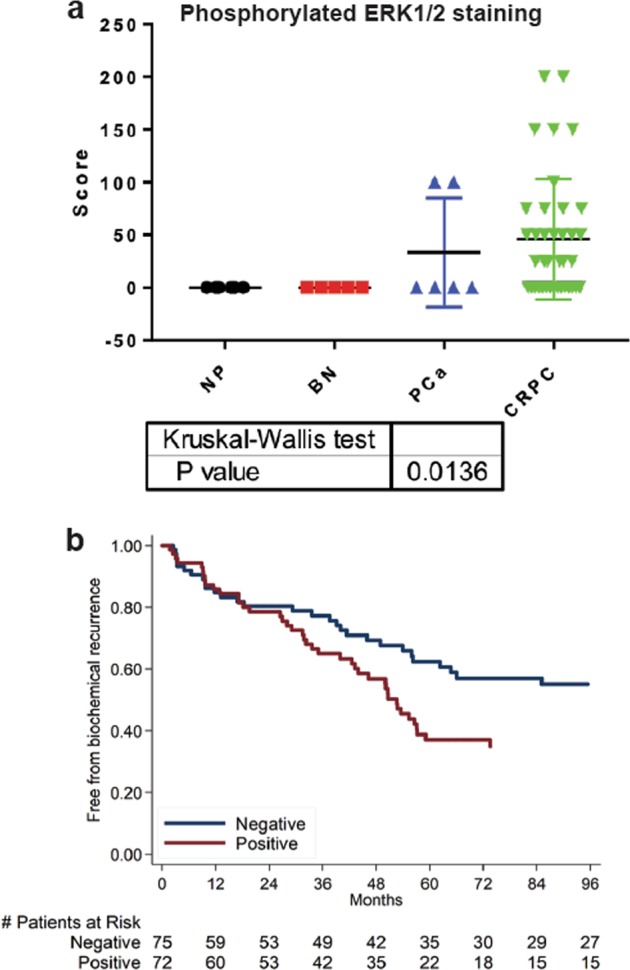
**a** Cytoplasmic epithelial staining for phosphorylated ERK1/2. NP normal gland, BN benign glands adjacent to primary prostate cancer, PCa untreated primary prostate cancer, CRPC castration resistant prostate cancer. **b** Absence (negative) or presence (positive) of ERK1/2 phosphorylation within resected primary prostate cancer. Recurrence is associated with ERK1/2 phosphorylation

Prior reports demonstrated that ERK1 and 2 are phosphorylated in mCRPC at high frequencies [8, 9]. Phosphorylation of residues Thr202 and Tyr204 was evaluated as detected by immunohistochemistry on tissue comprised of normal prostate tissue controls, localized primary prostate cancer, benign tissue adjacent to the primary prostate cancer, and CRPC. Epithelial staining of phosphorylated ERK1/2 was significantly higher in CRPC compared to primary prostate tumors and benign tissue (Fig. 2a). Given the difference in ERK hyperactivation in CRPC compared to primary prostate cancer tissue, we postulated that ERK1/2 activation in primary tumors correlates with risk for biochemical failure after radical prostatectomy. TMAs consisting of 147 radical prostatectomy specimens from patients with localized prostate cancer and a median of 7.2 years follow-up were stained for phosphorylated ERK1/2 (Table 1). Specimens were scored as positive or negative by immunohistochemistry. Phosphorylated ERK1/2 in the prostatectomy specimens was associated with biochemical recurrence, both adjusted (HR 1.66, *p* = 0.047) for clinicopathologic features (PSA, age, Gleason grade, race, year, margins status, T stage) and unadjusted (HR 1.79, *p* = 0.013) (Fig. 2b).

**Table 1:** Demographic, clinical, and pathological characteristics of patients that generated the TMAs

	(*N* = 147)
Age, M (Q1–Q3)	63 (58–67)
Race, n (%)	
Non-black	82 (56%)
Black	65 (44%)
Year of Surgery, M (Q1-Q3)	1998 (1994–1999)
PSA (ng/mL), M (Q1-Q3)	8.9 (5.6–13.4)
Pathological Grade Group, n (%):	
1	74 (50%)
2–3	67 (46%)
4–5	6 (4%)
Positive Margins, n (%)	58 (39%)
Seminal vesicle invasion, n (%)	16 (11%)
Extracapsular extension, n (%)	13 (9%)
Positive lymph nodes, n (%)	26 (18%)
Follow-up (years), M (Q1–Q3)	7.2 (6.2–8.7)

*SD* standard deviation, *M* median, *Q1* 25^th^ percentile, *Q3* 75^th^ percentile, *BMI* body mass index, *PSA* prostate specific antigen

**Fig. 3:**
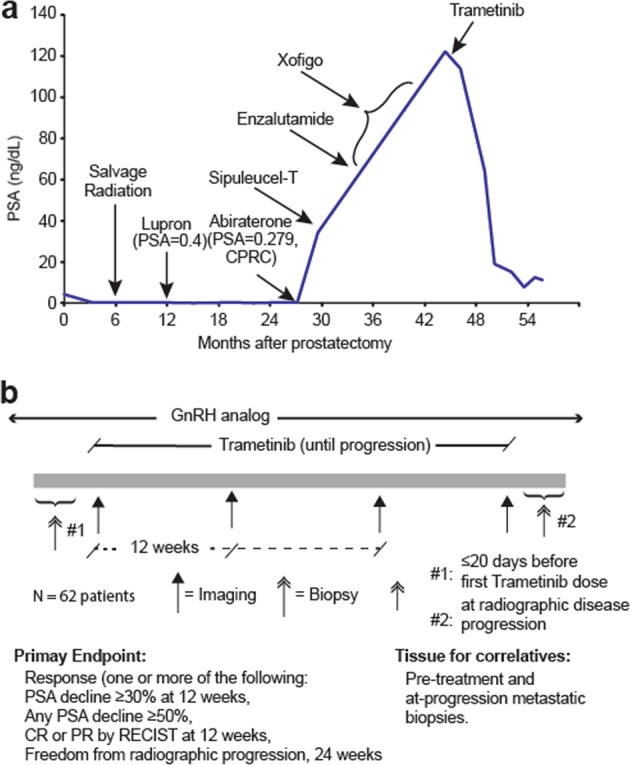
**a** A patient with mCRPC who had progressed on abiraterone, sipuleucel-T, enzalutamide, and radium-223 was treated with trametinib, which induced a PSA response of 85% at three and 93% at five months. **b** Schematic of ongoing proof of concept Phase II clinical trial of trametinib for patients with mCRPC who have progressed on one or more prior therapies for mCRPC (NCT02881242). Correlative analyses aim to identify patients most likely to respond and suggest possible pathways of resistance

A patient with mCRPC complicated by transfusion dependent anemia who had previously progressed on abiraterone, sipuleucel-T, enzalutamide, and radium-223, was initiated on trametinib therapy at 2 mg daily and had a serum PSA reduction of 85 and 93% at three and five months, respectively (Fig. 3a). His hemoglobin stabilized and he was no longer transfusion dependent after initiation of trametinib. The patient remained on trametinib without radiographic or clinical progression until the patient experienced a lethal stroke approximately 18 months after treatment initiation. A bone biopsy prior to treatment initiation in this patient did not yield tissue sufficient for targeted gene or RNA sequencing.

## Discussion

Sequencing of mCRPCs has not revealed frequent recurrent gain-of-function mutations in kinases, including the MAP kinases [2, 3]. However, our data suggest that ERK1/2 may be a potential kinase target in mCRPCs based on the clinical proteomic and transcriptomic data. Taken alone, the ERK activation signature inferred from the transcriptome of mCRPC by VIPER analysis could be explained by mechanisms independent of ERK itself. For example, Ets variant transcription factors can activate a MAPK transcriptional program in the absence of ERK activation in prostate cancer cell models [18]. However, the clinical phosphoproteomic data demonstrates intense and frequent phosphorylation of ERK1/2 in mCRPC and is consistent with direct activation of this kinase.

To our knowledge, the overall frequency of 32% that we report for amplifications of MAPK pathway members within mCRPCs has not previously been reported. Importantly, this observed frequency of amplifications of these genes does not imply a proximal mechanism of activation for MAPK activation within mCRPCs. Notably, prior publications have reported increased expression of MAPK pathway members [8, 9, 19, 20] and high levels of phosphorylated ERK1/2 within mCRPCs [8]. Compensatory activation of PI3 kinase and MAPK can occur in the context of suppressed androgen receptor signaling [8, 21]. Mechanistic studies in models of castration resistant, AR null prostate cancers demonstrate hyperactive MAPK signaling activated by paracrine and autocrine FGF/FGFR activation [16]. AR null prostate cancer xenografts were also shown to be sensitive to inhibitors of MAPK or FGFR [16].

Our finding that CRPC tissue has phosphorylated ERK1/2 far exceeding that of most primary prostate tumors and benign prostate tissue is consistent with prior reports [8]. The association of ERK1/2 phosphorylation in the primary tumor with biochemical recurrence has not been previously reported. However, an earlier study of sixty-three primary prostate tumors found a positive correlation between ERK1/2 phosphorylation and both T stage and Gleason Grade [22], and with rapid progression to CRPC [23].

A large number of kinase inhibitors have been tested in clinical trials for mCRPC, including dasatanib (multiple targets including SRC) [24], cabozantinib (MET and VEGFR2) [25], buparlisib (PI3 kinase) [26], MLN0128 (mTOR) [27], and sorafenib (multiple targets including RAF) [28], with largely disappointing results [29]. Given these prior negative trials of single agent kinase inhibitors in combination with hormone suppression, any prospective trial of yet another kinase inhibitor for patients with mCRPC is approached with cautious optimism at best.

Prior studies in the PTEN deletion mouse model system found overexpression of members of the MAPK signaling pathway ARAF, BRAF, and CRAF (along with MERTK and NTRK2) promotes metastases [5]. Positive staining for these kinases in 69%, 15%, and 26% in mCRPC suggests these kinases may also be viable targets [5]. However, the multi-target kinase inhibitor sorafenib, which inhibits BRAF and CRAF, performed unimpressively in Phase II trials in mCRPC [28, 30]. Targeting the MAPK downstream, for example MEK1/2 or ERK, may be more successful than BRAF or CRAF due to activation of ERK signaling by RAF inhibitors in the context of wild-type BRAF [31], which is a characteristic of most mCRPCs [2, 3].

## Conclusion

Our data support an ongoing single-arm proof of concept phase II study of single agent trametinib in men with mCRPC who have progressed on at least one prior line of therapy for mCRPC that includes abiraterone and/or enzalutamide (Fig. 3b). ERK1 activation status may represent a potential molecular selection marker for inclusion on this study; however, the fact that the most mCRPC specimens manifest increased ERK activation suggests that factors independent of ERK activation status may predict for response to trametinib. It is possible that signaling redundancies and activation of reciprocal pathways may frustrate or limit efficacy of MEK inhibition alone, potentially necessitating co-targeting of additional pathways activated in mCRPC, a strategy that has been explored in model systems (Mulholland et al.). Exomic and transcriptomic analyses of study biopsies performed at baseline and at the time of progression will facilitate our understanding of the biomarkers of response and resistance to trametinib, and inform future trials of MEK inhibitors for mCRPC.

## Supplementary information

Supplemental Table 1

Supplemental Table 2

## References

[1] Gross S, Rahal R, Stransky N, Lengauer C, Hoeflich KP (2015). Targeting cancer with kinase inhibitors. J Clin Invest.

[2] Quigley DA, Dang HX, Zhao SG, Lloyd P, Aggarwal R, Alumkal JJ (2018). Genomic hallmarks and structural variation in metastatic prostate cancer. Cell.

[3] Robinson D, Van Allen EM, Wu YM, Schultz N, Lonigro RJ, Mosquera JM (2015). Integrative clinical genomics of advanced prostate cancer. Cell.

[4] Drake JM, Graham NA, Stoyanova T, Sedghi A, Goldstein AS, Cai H (2012). Oncogene-specific activation of tyrosine kinase networks during prostate cancer progression. Proc Natl Acad Sci USA.

[5] Faltermeier CM, Drake JM, Clark PM, Smith BA, Zong Y, Volpe C (2016). Functional screen identifies kinases driving prostate cancer visceral and bone metastasis. Proc Natl Acad Sci USA.

[6] Drake JM, Paull EO, Graham NA, Lee JK, Smith BA, Titz B (2016). Phosphoproteome integration reveals patient-specific networks in prostate. Cancer Cell.

[7] Aggarwal R, Huang J, Alumkal JJ, Zhang L, Feng FY, Thomas GV (2018). Clinical and genomic characterization of treatment-emergent small-cell neuroendocrine prostate cancer: a multi-institutional prospective study.. J Clin Oncol Off J Am Soc Clin Oncol.

[8] Mulholland DJ, Kobayashi N, Ruscetti M, Zhi A, Tran LM, Huang J (2012). Pten loss and RAS/MAPK activation cooperate to promote EMT and metastasis initiated from prostate cancer stem/progenitor cells. Cancer Res.

[9] Taylor BS, Schultz N, Hieronymus H, Gopalan A, Xiao Y, Carver BS (2010). Integrative genomic profiling of human prostate cancer. Cancer Cell.

[10] Alvarez MJ, Shen Y, Giorgi FM, Lachmann A, Ding BB, Ye BH (2016). Functional characterization of somatic mutations in cancer using network-based inference of protein activity. Nat Genet.

[11] Newton Y, Novak AM, Swatloski T, McColl DC, Chopra S, Graim K (2017). TumorMap: exploring the molecular similarities of cancer samples in an interactive portal. Cancer Res.

[12] Hirsch FR, Varella-Garcia M, Bunn PA, Di Maria MV, Veve R, Bremmes RM (2003). Epidermal growth factor receptor in non-small-cell lung carcinomas: correlation between gene copy number and protein expression and impact on prognosis. J Clin Oncol J Am Soc Clin Oncol.

[13] Araujo JC, Trudel GC, Saad F, Armstrong AJ, Yu EY, Bellmunt J (2013). Docetaxel and dasatinib or placebo in men with metastatic castration-resistant prostate cancer (READY): a randomised, double-blind phase 3 trial. Lancet Oncol.

[14] Roberts PJ, Der CJ (2007). Targeting the Raf-MEK-ERK mitogen-activated protein kinase cascade for the treatment of cancer. Oncogene.

[15] Samatar AA, Poulikakos PI (2014). Targeting RAS-ERK signalling in cancer: promises and challenges. Nat Rev Drug Discov.

[16] Bluemn EG, Coleman IM, Lucas JM, Coleman RT, Hernandez-Lopez S, Tharakan R (2017). Androgen receptor pathway-independent prostate cancer is sustained through FGF signaling. Cancer Cell.

[17] Buscà R, Pouysségur J, Lenormand P (2016). ERK1 and ERK2 map kinases: specific roles or functional redundancy?. Front Cell Dev Biol.

[18] Hollenhorst PC, Ferris MW, Hull MA, Chae H, Kim S, Graves BJ (2011). Oncogenic ETS proteins mimic activated RAS/MAPK signaling in prostate cells. Genes Dev.

[19] Feng S, Shao L, Castro P, Coleman I, Nelson PS, Smith PD (2017). Combination treatment of prostate cancer with FGF receptor and AKT kinase inhibitors. Oncotarget.

[20] Armstrong K, Ahmad I, Kalna G, Tan SS, Edwards J, Robson CN (2011). Upregulated FGFR1 expression is associated with the transition of hormone-naive to castrate-resistant prostate cancer. Br J Cancer.

[21] Carver BS, Chapinski C, Wongvipat J, Hieronymus H, Chen Y, Chandarlapaty S (2011). Reciprocal feedback regulation of PI3K and androgen receptor signaling in PTEN-deficient prostate cancer. Cancer Cell.

[22] Gioeli D, Mandell JW, Petroni GR, Frierson HF, Weber MJ (1999). Activation of mitogen-activated protein kinase associated with prostate cancer progression. Cancer Res.

[23] Mukherjee R, McGuinness DH, McCall P, Underwood MA, Seywright M, Orange C (2011). Upregulation of MAPK pathway is associated with survival in castrate-resistant prostate cancer. Br J Cancer.

[24] Twardowski PW, Beumer JH, Chen CS, Kraft AS, Chatta GS, Mitsuhashi M (2013). A phase II trial of dasatinib in patients with metastatic castration-resistant prostate cancer treated previously with chemotherapy. Anticancer Drugs.

[25] Smith M, De Bono J, Sternberg C, Le Moulec S, Oudard S, De Giorgi U (2016). Phase III study of cabozantinib in previously treated metastatic castration-resistant prostate cancer: COMET-1. J Clin Oncol J Am Soc Clin Oncol.

[26] Armstrong AJ, Halabi S, Healy P, Alumkal JJ, Winters C, Kephart J (2017). Phase II trial of the PI3 kinase inhibitor buparlisib (BKM-120) with or without enzalutamide in men with metastatic castration resistant prostate cancer. Eur J Cancer Oxf Engl 1990.

[27] Graham L, Banda K, Torres A, Carver BS, Chen Y, Pisano K (2018). A phase II study of the dual mTOR inhibitor MLN0128 in patients with metastatic castration resistant prostate cancer. Invest New Drugs.

[28] Aragon-Ching JB, Jain L, Gulley JL, Arlen PM, Wright JJ, Steinberg SM (2009). Final analysis of a phase II trial using sorafenib for metastatic castration-resistant prostate cancer. BJU Int.

[29] Limvorasak S, Posadas EM (2009). Kinase inhibitors in prostate cancer. Anticancer Agents Med Chem.

[30] Beardsley EK, Hotte SJ, North S, Ellard SL, Winquist E, Kollmannsberger C (2012). A phase II study of sorafenib in combination with bicalutamide in patients with chemotherapy-naive castration resistant prostate cancer. Invest New Drugs.

[31] Poulikakos PI, Zhang C, Bollag G, Shokat KM, Rosen N (2010). RAF inhibitors transactivate RAF dimers and ERK signalling in cells with wild-type BRAF. Nature.

