# Supplementary material for: MEK-ERK signaling is a therapeutic target in metastatic castration resistant prostate cancer
Source: Prostate Cancer Prostatic Dis. 2019 Feb 25;22(4):531–8. doi: 10.1038/s41391-019-0134-5 (PMC6853839; doi:10.1038/s41391-019-0134-5)
Supplement: Supplementary file 1 — Supplemental Table 1 [file 41391_2019_134_MOESM1_ESM.docx]

**Supplementary Table 1: VIPER scores (mCRPC / SU2C vs localized / TCGA).** NES: normalized enrichment score.

| **Kinase** | **NES** | **p.value** |
| --- | --- | --- |
| PRKACA | 3.043238522 | 0.002340467 |
| GSK3A | 2.180726012 | 0.029203688 |
| PRKCD | 2.17757092 | 0.029437996 |
| MAPK3 | 2.132603221 | 0.032957289 |
| MAPKAPK2 | 2.062912948 | 0.0391209 |
| SRC | 2.060146669 | 0.039384521 |
| CSNK2A1 | 1.966860168 | 0.049199334 |
| JAK2 | 1.811182511 | 0.070112605 |
| MAPK9 | 1.754232007 | 0.079390761 |
| PRKD1 | 1.635823152 | 0.101876605 |
| RPS6KA1 | 1.531886864 | 0.125550356 |
| PRKCZ | 1.368868825 | 0.171040282 |
| AKT2 | 1.166407569 | 0.2434497 |
| EGFR | 1.165012896 | 0.244013773 |
| ABL1 | 1.14996774 | 0.250157159 |
| RPS6KA3 | 1.132332905 | 0.257494501 |
| PRKCE | 1.112107321 | 0.266092009 |
| CDK5 | 1.00209427 | 0.316298065 |
| PDPK1 | 0.920581296 | 0.357269071 |
| FYN | 0.920485351 | 0.357319185 |
| MTOR | 0.866051723 | 0.386461798 |
| GSK3B | 0.85469846 | 0.392718103 |
| INSR | 0.845423978 | 0.397874162 |
| LCK | 0.708351181 | 0.478727201 |
| MAPK14 | 0.67495128 | 0.499706719 |
| PRKAA1 | 0.651773931 | 0.514547021 |
| AKT1 | 0.632197103 | 0.52725809 |
| CSNK2A2 | 0.515425272 | 0.606255876 |
| AURKA | 0.482058489 | 0.629764397 |
| PRKDC | 0.387289595 | 0.698541828 |
| PAK1 | 0.373145328 | 0.709040284 |
| PRKCA | 0.113331185 | 0.909767995 |
| RPS6KB1 | 0.061071163 | 0.951302535 |
| SYK | -0.026075387 | 0.979197209 |
| LYN | -0.038874577 | 0.968990386 |
| PRKG1 | -0.167780135 | 0.866756251 |
| MAPK8 | -0.185157121 | 0.8531058 |
| CHEK2 | -0.303193301 | 0.761742554 |
| PRKCB | -0.394873403 | 0.692936319 |
| CAMK2A | -0.421145331 | 0.673648962 |
| IKBKB | -0.469459231 | 0.63874142 |
| MAPK1 | -0.632795458 | 0.526867223 |
| SGK1 | -0.649515827 | 0.51600502 |
| ROCK1 | -0.779634358 | 0.435606126 |
| CSNK1A1 | -0.794906304 | 0.426668007 |
| AURKB | -1.342595815 | 0.179402886 |
| CDK1 | -1.670366129 | 0.094846947 |
| ATR | -1.729377832 | 0.083741495 |
| PRKCQ | -1.751105484 | 0.079927742 |
| PLK1 | -1.963593539 | 0.049577247 |
| CHEK1 | -2.424415639 | 0.015333045 |
| CDK2 | -3.469857214 | 0.000520735 |
| ATM | -4.414752498 | 1.01E-05 |
