# Supplementary material for: MEK-ERK signaling is a therapeutic target in metastatic castration resistant prostate cancer
Source: Prostate Cancer Prostatic Dis. 2019 Feb 25;22(4):531–8. doi: 10.1038/s41391-019-0134-5 (PMC6853839; doi:10.1038/s41391-019-0134-5)
Supplement: Supplementary file 2 — Supplemental Table 2 [file 41391_2019_134_MOESM2_ESM.docx]

**Supplementary Table 2: ERK1/2 phosphorylation substrates**

| **Mean Difference (Log10)** | **Phosphosite** | **Gene Name** |
| --- | --- | --- |
| 1.567565091 | pS216 | HNRNPK |
| 1.488629076 | pS11 | RCC1 |
| 1.317320342 | pS272, pS275 | SQSTM1 |
| 1.211248422 | pS4, pS10 | NPM1 |
| 1.206203597 | pS183 | EIF3H |
| 1.073311317 | pS70 | NPM1 |
| 1.071533755 | pS40, pS41 | MCM2 |
| 1.024342425 | pS27 | MCM2 |
| 1.022002491 | pS682, pT687 | RPS6KA4 |
| 1.006206622 | pT221 | RPS3 |
| 0.975463634 | pS502, pS514 | NOP58 |
| 0.932020965 | pS587 | EIF4ENIF1 |
| 0.846226075 | pS711, pT722 | MCM3 |
| 0.823038031 | pS328 | RAD9A |
| 0.814888935 | pT359 | ECT2 |
| 0.797339223 | pS308 | AR |
| 0.686143692 | pS639 | BCAR1 |
| 0.652916984 | pS47 | SIRT1 |
| 0.649326845 | pS314, pS326 | HSF1 |
| 0.645961228 | pS1213 | TOP2A |
| -0.657932892 | pS221 | NUP50 |
| -0.683265724 | pS38 | STMN1 |
| -0.71293507 | pS100 /// pS73 | JUN |
| -0.75572765 | pS447 /// pS1053 | RPS6KB1 |
| -0.769170858 | pS670 | ADRBK1 |
| -0.830179469 | pS857 | NCOA3 |
| -0.857412366 | pS259 | PIK3C2A |
| -0.868377524 | pT509 | CRMP1 |
| -0.906663681 | pS41 | CARHSP1 |
| -0.930106084 | pS126, pS130 | PXN |
| -1.000990392 | pS63 | JUN |
| -1.419013637 | pS8 | PARVA |
| -1.882126312 | pT51 | PAGE4 |
| -2.039626451 | pS789 | CALD1 |
